# Risk Factors Related to the Incidence of Stroke-Associated Pneumonia in Adults: Protocol for a Scoping Review

**DOI:** 10.2196/90248

**Published:** 2026-07-15

**Authors:** Ana Karolina Ibanhes, Pryscilla Kathiana Maia Freitas da Silveira Redigolo, Anna Alicia Pereira Duarte, Anna Letícia Pereira Duarte, Carolina Mariano Pompeo

**Affiliations:** 1 Universidade Federal de Mato Grosso do Sul Campo Grande, Mato Grosso do Sul Brazil

**Keywords:** stroke, stroke-associated pneumonia, risk factors, nursing, critical care nursing, evidence synthesis, scoping review, pneumonia prevention, poststroke complications, hospital care, adult patients, infection control

## Abstract

**Background:**

Cerebrovascular diseases represent a major public health challenge, and stroke is among the leading causes of mortality worldwide. Among poststroke complications, pneumonia stands out because of its frequency and negative impact on clinical outcomes, including prolonged hospitalization and increased mortality. In this context, studies investigating the risk factors associated with stroke-related pneumonia differ in terms of their design, care setting, and adopted definitions.

**Objective:**

This study aims to map risk factors related to the incidence of pneumonia in adults hospitalized after stroke.

**Methods:**

This scoping review protocol was developed in accordance with the *JBI Reviewer’s Manual* and the PRISMA-ScR (Preferred Reporting Items for Systematic Reviews and Meta-Analyses extension for Scoping Reviews). Searches will include the following indexed databases: PubMed (MEDLINE), Embase, Scopus, the Cochrane Library, Web of Science, and the Virtual Health Library. Gray literature will be searched in Google Scholar, the CAPES Theses and Dissertations Catalog, the Brazilian Digital Library of Theses and Dissertations, ProQuest, SciELO Preprints, medRxiv, ClinicalTrials.gov, and the Brazilian Registry of Clinical Trials. Additional organizational sources will include the World Health Organization, the Pan American Health Organization, the Centers for Disease Control and Prevention, the European Stroke Organisation, and the Brazilian Ministry of Health. Qualitative, quantitative, and mixed methods studies, including observational and experimental designs, will be considered, with no language or time restrictions, provided that they meet the eligibility criteria defined in the protocol. Study selection will follow 3 stages using Mendeley (Elsevier) and Rayyan (Rayyan Systems Inc).

**Results:**

This protocol was funded in June 2026 by the Federal University of Mato Grosso do Sul and the Coordination for the Improvement of Higher Education Personnel (Finance Code 001). The protocol was developed and prospectively registered in the Open Science Framework. Preliminary searches were carried out in August 2025 in PubMed (MEDLINE), Embase, and the Cochrane Library to test the sensitivity of the search strategies and estimate the potential volume of eligible studies. At the time of publication, the final search, study selection, data extraction, and evidence synthesis had been completed. The manuscript reporting the final review results is expected to be submitted for publication in early 2027.

**Conclusions:**

This review is expected to contribute to the systematization of evidence on risk factors related to stroke-associated pneumonia, identify knowledge gaps, and support future prevention strategies and clinical management of hospitalized patients.

**Trial Registration:**

Open Science Framework 10.17605/OSF.IO/EXYWZ; https://osf.io/exywz/overview

**International Registered Report Identifier (IRRID):**

PRR1-10.2196/90248

## Introduction

### Background

Cerebrovascular diseases constitute an important public health problem and remain among the leading causes of morbidity and mortality worldwide. Stroke accounts for millions of cases annually and ranks prominently among the leading causes of death worldwide, as reported by the World Health Organization [1]. In addition to high mortality, stroke is also associated with prolonged hospitalization and important functional repercussions during care [2].

Among the clinical complications resulting from stroke, pneumonia stands out because of its frequency and negative impact on patient outcomes [3-5]. Studies and literature reviews have shown an association between poststroke pneumonia and increased mortality, longer hospital stays, and poorer functional recovery [4,5]. Therefore, recognizing its risk factors is of both clinical and care-related relevance.

The literature has already described different factors related to the occurrence of pneumonia after stroke, such as greater neurological severity, dysphagia, decreased level of consciousness, mechanical ventilation, use of a nasogastric tube, malnutrition, previous comorbidities, and inflammatory markers [4,6-16]. Nevertheless, available studies differ in terms of their methodological designs, care settings, diagnostic definitions of pneumonia, and the way risk factors are reported [4-7,16]. This variability makes comparisons across findings difficult and limits an integrated understanding of the topic.

In this context, a scoping review is appropriate for gathering and organizing the available studies on the topic. According to the Joanna Briggs Institute (JBI), this type of review makes it possible to describe the existing scientific literature, identify knowledge gaps, and guide future investigations [17,18].

### Rationale

Despite the existence of primary studies and systematic reviews on stroke-associated pneumonia, the literature remains fragmented regarding the reported risk factors, methodological designs, care settings, and diagnostic definitions adopted [4-7,16]. In this sense, a scoping review does not aim to replace primary studies but rather to provide a structured mapping of the available evidence. In addition to synthesizing the knowledge produced on the topic, this review may support the planning of future investigations by helping to define variables and develop data collection instruments for subsequent observational studies.

### Objective

This study aims to map risk factors related to the incidence of pneumonia in adults hospitalized after stroke.

## Methods

### Design and Guidelines

This is a scoping review protocol conducted in accordance with the methodological guidance provided in the *JBI Reviewer’s Manual* [17]. The development and reporting of this protocol follow the recommendations of the PRISMA-ScR (Preferred Reporting Items for Systematic Reviews and Meta-Analyses extension for Scoping Reviews) [19] to ensure transparency, methodological rigor, and reproducibility.

The protocol was previously registered on the Open Science Framework, which ensures the public availability of the planned methods and helps prevent unnecessary duplication of reviews.

### Research Question

The research question is described in Textbox 1. It was structured based on the population, concept, and context strategy, as recommended by the JBI.

**Population, concept, and context (PCC) strategy.**

- Population: hospitalized adults (aged ≥18 years) with a confirmed stroke diagnosis, regardless of stroke subtype (ischemic or hemorrhagic)
- Concept: stroke-associated pneumonia, including aspiration pneumonia and ventilator-associated pneumonia, provided that the pneumonia is temporally related to the cerebrovascular event
- Context: hospital settings, including emergency services, hospital wards, and intensive care units

For the purposes of this review, the outcome of interest will be treated as stroke-related pneumonia in hospitalized adults after stroke. When studies specifically distinguish stroke-associated pneumonia, aspiration pneumonia, or ventilator-associated pneumonia, these terms will be extracted as originally reported by the authors. During study selection and data extraction, these outcomes will be grouped under the broader review concept only when they are explicitly related to the stroke event.

On the basis of this structure, the following guiding question was formulated: What are the risk factors related to the incidence of pneumonia in adults hospitalized after stroke?

### Eligibility Criteria

The eligibility criteria were defined in advance based on the research question and the population, concept, and context strategy. Textbox 2 presents the inclusion and exclusion criteria adopted for this review.

**Inclusion and exclusion criteria.**

**Inclusion criteria**

- Studies involving hospitalized adults (aged ≥18 years) with a confirmed stroke diagnosis, regardless of stroke subtype (ischemic or hemorrhagic)
- Studies investigating the occurrence of pneumonia after stroke and reporting the incidence of pneumonia and/or factors associated with its development
- Studies using qualitative, quantitative, or mixed methods
- Experimental, quasi-experimental, and observational studies, including clinical trials, cohort studies, case-control studies, and cross-sectional studies
- Relevant gray literature, including clinical guidelines, institutional documents, technical manuals, dissertations, and theses
- Studies published in any language and during any period

**Exclusion criteria**

- Publications that do not explicitly address the relationship between stroke and pneumonia as an outcome or clinical complication
- Studies whose primary focus does not allow the identification or extraction of risk factors related to the incidence of pneumonia after stroke
- Case reports, letters to the editor, editorials, comments, expert opinions, and conference abstracts
- Duplicate versions of the same study, for which only the most complete or most recent publication will be considered

### Information Sources

The search will be conducted in indexed electronic databases and gray literature sources to ensure broad coverage of the available evidence. Indexed databases will include PubMed (MEDLINE), Embase, Scopus, the Cochrane Library, Web of Science, and the Virtual Health Library.

Gray literature will be searched through Google Scholar, the CAPES Theses and Dissertations Catalog, BDTD, ProQuest Dissertations and Theses, SciELO Preprints, medRxiv, ClinicalTrials.gov, and ReBEC. For this review, gray literature corresponds to documents not controlled by commercial scientific publishers. When retrieval exceeds a large number of records, screening will be limited to the first 100 results sorted by relevance in Google Scholar, ProQuest, and medRxiv.

In addition, relevant organizational sources will be considered, namely, the World Health Organization, the Pan American Health Organization, the Centers for Disease Control and Prevention, the European Stroke Organisation, and the Brazilian Ministry of Health. For these sources, identification of potentially relevant documents will be based on references and institutional mentions found in the included studies.

### Search Strategy

The search strategy was structured using controlled descriptors from the MeSH (Medical Subject Headings) and Emtree vocabularies, combined with free-text terms related to stroke, pneumonia, incidence, and risk factors. In August 2025, a total of 2 preliminary combinations were tested to assess search sensitivity and estimate the potential volume of eligible studies:

- Combination 1: stroke OR “cerebrovascular accident” AND pneumonia AND incidence AND “risk factors” AND adult
- Combination 2: stroke OR “brain vascular accident” AND pneumonia AND incidence AND “risk factors”

The tests were carried out in PubMed (MEDLINE), Embase, and the Cochrane Library, retrieving 2399, 806, and 1330 records, respectively. In these initial combinations, the purpose was to explore the behavior of the main descriptors related to the phenomenon of interest.

On the basis of this process, the strategy was refined to improve specificity and reproducibility. In the final search strategies, the adult population will be explicitly included, in line with the review question and the eligibility criteria established in the protocol. As an example, Textbox 3 presents the complete refined strategies for PubMed (MEDLINE) and Embase. The strategies for the other databases will be adapted according to their specific requirements.

**Refined search strategies for PubMed (MEDLINE) and Embase.**

**PubMed (MEDLINE)**

(“Stroke”[Mesh] OR stroke[tiab] OR “cerebrovascular accident*”[tiab] OR “ischemic stroke”[tiab] OR “hemorrhagic stroke”[tiab])

AND (“Pneumonia”[Mesh] OR pneumonia[tiab] OR “stroke-associated pneumonia”[tiab] OR “post-stroke pneumonia”[tiab] OR “aspiration pneumonia”[tiab])

AND (“Risk Factors”[Mesh] OR “risk factor*”[tiab] OR predictor*[tiab] OR determinant*[tiab])

AND ( “Adult”[Mesh] OR adult*[tiab])

**Embase**

(‘stroke’/mj OR stroke:ti,ab OR ‘cerebrovascular accident*’:ti,ab OR ‘ischemic stroke’:ti,ab OR ‘hemorrhagic stroke’:ti,ab)

AND (‘pneumonia’/mj OR pneumonia:ti,ab OR ‘stroke associated pneumonia’:ti,ab OR ‘post stroke pneumonia’:ti,ab OR ‘aspiration pneumonia’:ti,ab)

AND (‘risk factor’/de OR ‘risk factor*’:ti,ab OR predictor*:ti,ab OR determinant*:ti,ab)

AND (‘adult’/de OR adult*:ti,ab)

For gray literature, Google Scholar will be searched using the terms “stroke” AND “pneumonia” AND “risk factors” AND adults, and the first 100 results sorted by relevance will be screened. The same limit of screening the first 100 results will be applied to ProQuest Dissertations and Theses and medRxiv when retrieval exceeds this number. Searches in ClinicalTrials.gov will use the terms “stroke” AND “pneumonia” AND “risk factors,” while searches in ReBEC will use the terms “acidente vascular cerebral” AND pneumonia. The CAPES Theses and Dissertations Catalog and BDTD will be searched using terms aligned with the review question in Portuguese. For organizational sources, potentially relevant documents will be identified through references and institutional mentions found in the included studies.

### Study Selection and Data Extraction

All identified records will be exported to Mendeley (Elsevier) for reference management and duplicate removal. After this stage, the studies will be imported into the Rayyan (Rayyan Systems Inc) platform [20]. Selection will occur in 3 stages. In the first stage, titles and abstracts will be screened independently and blindly by AKI, PKMFDSR, AAPD, and ALPD based on the eligibility criteria. In the second stage, potentially eligible studies will be assessed in full text by the same reviewers. Finally, in the third stage, the final inclusion of studies will be determined through consensus and, when necessary, by CMP. The entire selection process will be described and presented using a PRISMA-ScR flow diagram.

Data extraction will be conducted using a standardized form developed based on the *JBI Reviewer’s Manual* and adapted to the objectives of the review. Before final extraction, the instrument will be tested on a sample of the included studies, allowing adjustments for greater clarity and consistency. The following information will be extracted:

- Full reference (authors, title, and year of publication)
- Study language
- Country or region where the study was conducted
- Type of publication
- Objectives and methodological design
- Characteristics of the studied population (age, sex, and stroke subtype)
- Diagnostic criteria for stroke-associated pneumonia
- Terminology used by the study to describe the pneumonia outcome (eg, stroke-associated pneumonia, aspiration pneumonia, and ventilator-associated pneumonia)
- Incidence of pneumonia
- Identified risk factors
- Type of association measure reported (eg, crude or adjusted odds ratio, risk ratio, hazard ratio, mean difference, or frequency measure)
- Whether the reported factor was presented as an unadjusted association or an adjusted predictor in multivariable analysis
- Associated clinical outcomes
- Limitations reported by the authors
- Additional relevant observations

### Data Synthesis and Presentation

The extracted data will be organized descriptively in tables and figures, accompanied by a narrative synthesis. Initially, a table will be developed presenting the characteristics of the included studies, including methodological design, country, care setting, population characteristics, stroke subtype, diagnostic criteria for pneumonia, and investigated risk factors.

The studies will also be described according to their methodological designs and the definitions used for stroke-associated pneumonia to highlight the differences among the contexts in which the evidence was produced and to improve the comparability of findings. This organization will allow the identification of the extent to which conceptual and methodological variations influence the way risk factors are described.

Risk factors will be grouped into thematic categories according to conceptual similarity. Expected groups include demographic and baseline clinical factors, neurological factors, dysphagia- and aspiration-related factors, care-related factors, and laboratory or nutritional factors. The narrative synthesis will seek to describe patterns, convergences, inconsistencies, and gaps in the literature without performing a meta-analysis or a pooled effect estimation.

The synthesis will preserve the original terminology adopted in the included studies and will describe whether pneumonia was reported as stroke-associated pneumonia, aspiration pneumonia, or ventilator-associated pneumonia. When these outcomes are explicitly related to stroke, they will be grouped under the broader review concept of stroke-related pneumonia, while conceptual distinctions will be described narratively.

When available, risk factors will be described separately according to whether they were reported as unadjusted associations or adjusted predictors from multivariable models. This distinction will be preserved in the tabular and narrative synthesis to improve the interpretability of the mapped evidence.

## Results

This scoping review protocol was developed, structured, and prospectively registered in the Open Science Framework to ensure methodological transparency and traceability of the planned stages. Preliminary searches were carried out in August 2025 in PubMed (MEDLINE), Embase, and the Cochrane Library to test the sensitivity of the search strategies and estimate the potential volume of eligible studies.

This protocol received institutional funding in June 2026 from the Federal University of Mato Grosso do Sul and the Coordination for the Improvement of Higher Education Personnel (Finance Code 001). At the time of publication, the final search strategies had been implemented, and study selection, data extraction, and evidence synthesis had been completed. The manuscript reporting the final review results is expected to be submitted for publication in early 2027.

**Figure 1:**
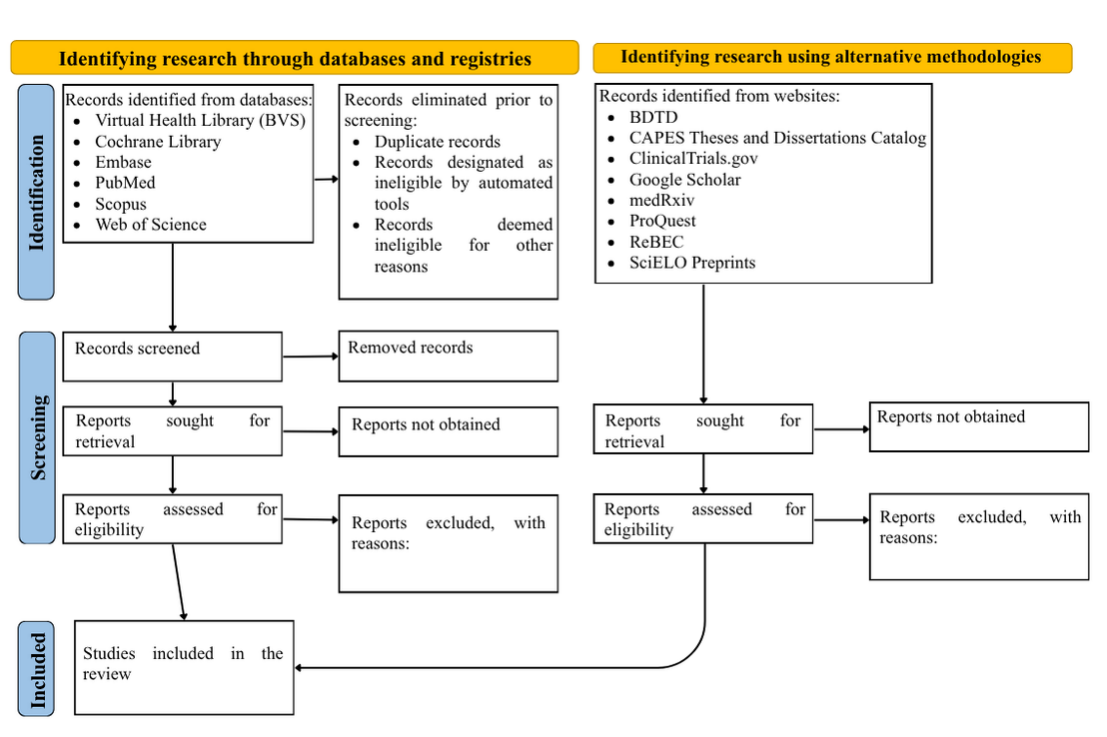
PRISMA-ScR flow diagram.

Figure 1 presents the PRISMA-ScR flow diagram used to represent the stages of identification, screening, eligibility, and inclusion of studies planned in this protocol.

## Discussion

### Anticipated Findings

Stroke-associated pneumonia is one of the most frequent and clinically relevant infectious complications in the context of hospitalization after a neurological event [3-5]. Observational studies and literature reviews indicate that this condition results from a complex interaction among clinical, neurological, functional, and health care–related factors [4,6-16]. Among the most frequently reported factors are older age, greater stroke severity, dysphagia, reduced level of consciousness, previous comorbidities, and exposure to invasive devices during hospitalization [4,6,11-15,21-30].

Given the methodological heterogeneity in study designs, populations, diagnostic definitions, and the reporting of risk factors in studies on this topic, a scoping review is methodologically appropriate because it allows the available literature to be gathered and organized, knowledge gaps to be identified, and the ways in which risk factors have been investigated to be described [17,18].

Adherence to the PRISMA-ScR recommendations in this protocol reinforces the commitment to transparency, standardized reporting, and methodological reproducibility, all of which are essential for review protocols [19]. The detailed description of the methodological stages will allow other researchers to understand, replicate, or update the review in the future.

The systematization of the risk factors described in the included studies may organize the available evidence into thematic categories, such as demographic, clinical, neurological, and care-related factors. This organization may contribute to the identification of potentially modifiable factors and support the development of preventive strategies and care protocols aimed at reducing the incidence of pneumonia in hospitalized patients after stroke [3-7].

From the perspective of clinical practice, especially in nursing and multiprofessional care, mapping risk factors may support clinical surveillance of more vulnerable patients, guide early interventions, and strengthen evidence-based preventive actions, such as swallowing assessment, early mobilization, oral hygiene, and appropriate management of invasive devices [3,4,8,11,30].

Although this review does not aim to assess the methodological quality or risk of bias of the included studies, in line with the nature of scoping reviews, a transparent description of the study designs and contexts will allow a critical interpretation of the findings and the identification of priority areas for future systematic reviews or primary studies [17-19].

Thus, this protocol proposes a methodologically rigorous approach to map risk factors associated with pneumonia in adults hospitalized after stroke, contributing to the consolidation of scientific knowledge and the improvement of health care, in line with international recommendations for the development and reporting of scoping review protocols [17-20].

### Study Limitations

This review has limitations that should be considered when interpreting its results. Because it is a scoping review, its primary objective is to map and organize the available literature rather than quantitatively estimate the strength of association between risk factors and stroke-associated pneumonia. Therefore, no formal assessment of the methodological quality or risk of bias of the included studies is planned.

Another expected limitation concerns the heterogeneity of the available literature. Included studies may differ in terms of their methodological designs, care settings, evaluated populations, and diagnostic definitions of stroke-associated pneumonia, which may limit direct comparability among findings. Differences in the way risk factors are reported are also expected, which may influence the organization and interpretation of the mapped evidence.

In addition, because this review will be descriptive and mapping oriented, no meta-analysis or quantitative pooling of results will be performed. Therefore, the findings should be interpreted as a broad synthesis of the available literature aimed at identifying patterns, gaps, and priority areas for future research.

## Data Availability

Materials related to this scoping review, including the registered protocol, the search strategies, the study selection flow diagram, the data extraction instrument, and the synthesis tables, will be publicly available in the Open Science Framework repository corresponding to the study. There are no primary data associated with this protocol.

## Abbreviations

JBI: Joanna Briggs Institute
MeSH: Medical Subject Headings
PRISMA-ScR: Preferred Reporting Items for Systematic Reviews and Meta-Analyses extension for Scoping Reviews
